# A Facile Co-Deposition Approach to Construct Functionalized Graphene Quantum Dots Self-Cleaning Nanofiltration Membranes

**DOI:** 10.3390/nano12010041

**Published:** 2021-12-23

**Authors:** Tong Yu, Chenpu Wu, Zhongyan Chen, Mingen Zhang, Zhuan Hong, Honghui Guo, Wenyao Shao, Quanling Xie

**Affiliations:** 1 Technology Innovation Center for Exploitation of Marine Biological Resources, Third Institute of Oceanography, Ministry of Natural Resources, Xiamen 361005, China; yutongtx_1@163.com (T.Y.); darrenwu.cp@foxmail.com (C.W.); 20420201151646@stu.xmu.edu.cn (Z.C.); 18859551701@163.com (M.Z.); zhong@tio.org.cn (Z.H.); hhguo@tio.org.cn (H.G.); 2 Department of Chemical and Biochemical Engineering, College of Chemistry and Chemical Engineering, Xiamen University, Xiamen 361005, China; 3 Fujian Collaborative Innovation Center for Exploitation and Utilization of Marine Biological Resources, Xiamen 361005, China

**Keywords:** nanofiltration, graphene quantum dots, L-aspartic acid, self-cleaning, co-deposition

## Abstract

In this study, a novel photocatalytic self-cleaning nanofiltration (NF) membrane was fabricated by constructing aspartic acid-functionalized graphene quantum dots (AGQDs) into the polydopamine/polyethyleneimine (PDA/PEI) selective layer via the co-deposition method. The chemical composition, microstructure, and hydrophilicity of the prepared membranes were characterized by scanning electron microscopy (SEM), atomic force microscopy (AFM), attenuated total reflection (ATR-FTIR), X-ray photoelectron spectroscopy (XPS), and water contact angle (WCA). Meanwhile, the effects of PEI molecular weight and AGQDs concentration on NF membrane structures and separation performance were systematically investigated. The photocatalytic self-cleaning performance of the PDA/PEI/AGQDs membrane was evaluated in terms of flux recovery rate. For constructing high-performance NF membranes, it is found that the optimal molecular weight of PEI is 10,000 Da, and the optimal concentration of AGQDs is 2000 ppm. The introduction of hydrophilic AGQDs formed a more hydrophilic and dense selective layer during the co-deposition process. Compared with the PDA/PEI membrane, the engineered PDA/PEI/AGQDs NF membrane has enhanced water flux (55.5 LMH·bar^−1^) and higher rejection (99.7 ± 0.3% for MB). In addition, the PDA/PEI/AGQDs membrane exhibits better photocatalytic self-cleaning performance over the PDA/PEI membrane (83% vs. 69%). Therefore, this study provides a facile approach to construct a self-cleaning NF membrane.

## 1. Introduction

The environmental hazards of dye wastewater should not be underestimated. Membrane separation technology has the advantage of energy-saving and high efficiency, which is widely applied to treat dye wastewater. However, membrane fouling is a critical issue limiting the application of membrane separation technology in dye treatment, which increases costs and reduces efficiency. Photocatalytic self-cleaning membranes provide a possible solution [1,2]. Photocatalytic self-cleaning membranes are commonly prepared by introducing nanoparticles with photocatalytic properties, which can degrade contaminants on the membrane surface by light irradiation [3,4,5,6]. On the one hand, the degradation of pollutants by light irradiation improves the efficiency of the membrane cleaning process. On the other hand, self-cleaning achieves membrane reuse, improves membranes’ anti-fouling performance, and reduces membrane loss and costs. More and more researchers are devoted to developing high-performance photocatalytic self-cleaning NF membranes.

Dopamine (DA), often referred to as “bio-glue”, is widely used for the surface functionalization of different substrate membranes [7]. Under alkaline conditions, dopamine is readily oxidized to form polydopamine (PDA) with strong adhesion [8], which enhances the stability of the membrane by strongly binding to the substrate. In addition, the unique chemical properties of PDA can act as a free-radical scavenger to protect the composite nanofiltration (NF) membrane [9,10]. Shao et al. [11] prepared self-cleaning NF membranes by constructing a PDA/GQDs interlayer using the interfacial polymerization method, and the fabricated NF membranes containing GQDs could effectively degrade organic molecules. The encapsulation of GQDs by the PDA/GQDs interlayer may play a crucial role in preventing the catalytic performance of GQDs from deactivating. However, PDA would easily be damaged under a strongly alkaline environment. At the same time, the long-term self-polymerization process of PDA on the membrane surface tends to block membrane pores [12], which is hindering the application of PDA in the membrane field.

Polyethyleneimine (PEI) can react with PDA through Michael addition or Schiff base reaction, thus mitigating the self-polymerization process of PDA on the membrane surface [13]. The co-deposition of PDA with PEI on the surface of polyphenylene sulfide (PPS) membranes facilitates the construction of NF membranes with high hydrophilicity, acid and alkaline resistance, and high dye separation [13]. Lv et al. [14] used a biomimetic mineralization method to grow β-FeOOH nanorods on a co-deposited PDA-PEI layer. Due to the strong coordination of Fe^3+^ and catechol groups, the PDA–PEI layer can serve as the selective layer for NF membranes and provide a spot to anchor the growth of β-FeOOH nanorods, resulting in the excellent self-cleaning property of the fabricated NF membrane. As a new type of zero-dimensional nanomaterial, graphene quantum dots (GQDs) also have the property of photocatalytic degradation of dyes [15,16]. Compared with β-FeOOH, GQDs with small sizes can form a more uniform membrane surface [11,17]. The functionalization of GQDs with aspartic acid is expected to improve the hydrophilicity and further enhance the photocatalytic performance of GQDs [18,19].

Herein, we fabricated a photocatalytic self-cleaning NF membrane by introducing AGQDs into the PDA/PEI composite layer via a green and environment-friendly co-deposition method. The chemical cross-linking of PDA and PEI enhanced the stability of the selective layer of the NF membrane [20]. AGQDs impart self-cleaning ability to the PDA/PEI/AGQDs membrane, which effectively degrades the enriched methyl blue molecules on the membrane surface. Furthermore, our work has optimized the molecular weight of PEI and determined the best introducing concentration of AGQDs in the PDA/PEI substrate, which would have a significant reference for dyes removal by NF membranes.

## 2. Materials and Methods

### 2.1. Materials

Citric acid (CA, 98% purity), caustic soda (NaOH, 99% purity), and hydrochloric acid (HCl, 37% purity) were purchased from Sinopharm Chemical Reagent Company (Shanghai, China). L-aspartic acid (>98% purity) and dopamine hydrochloride were purchased from Sigma-Aldrich Trading Co. Ltd. (Shanghai, China). PEI (Mw = 600 Da, Mw = 1800 Da, Mw = 10 kDa) was produced by Shanghai Macklin Biochemical Co., Ltd. (Shanghai, China). Methyl blue (MB, 1% purity), methyl orange (MO, 99% purity), Congo red (CR, 78% purity), and Rhodamine B (RhB, 99% purity) were purchased from Shanghai Aladdin (Shanghai, China). The polyethersulfone (PES, Mw = 10 kDa) substrate was provided by Shanghai Mosu Science Equipment Co., Ltd. (Shanghai, China).

### 2.2. Synthesis of AGQDs

**Figure 1:**
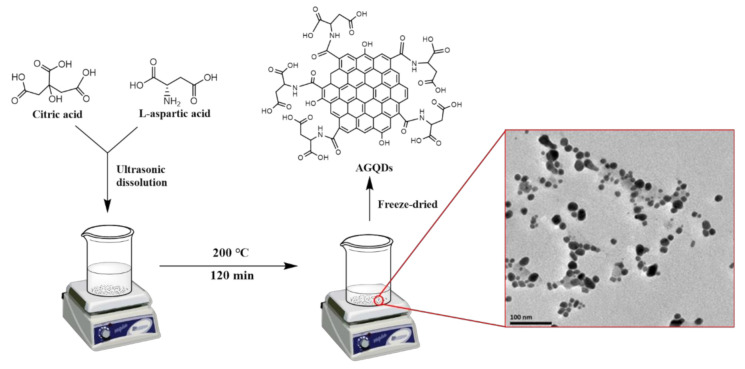
Scheme of the synthetic process of AGQDs.

As shown in Figure 1, AGQDs were synthesized by a one-step hydrothermal method [21]. Briefly, 10 g of CA and a certain amount of L-aspartic acid were mixed into 50 mL of deionized water. The mixture was ultrasonicated for 30 min to ensure a homogeneous mixture. Then, the mixture was heated at 200 °C for 120 min, during which time CA and L-aspartic acid reacted. The solution gradually changes from colorless to red–orange, indicating the formation of AGQDs. Subsequently, the solution was neutralized using 1 M NaOH. The neutralized solution was purified in a dialysis bag (1000 Da) for 24 h to remove unreacted reactants. Finally, the purified solution was freeze-dried to obtain AGQDs powder. The pristine GQDs were synthesized and purified by the same method except using CA as raw material.

### 2.3. Preparation of NF Membranes

The NF membranes were prepared by the co-deposition method using physical pressurization. The specific steps are as follows:

a. The polyethersulfone (PES) substrate was immersed in deionized water for 30 min to remove the protective solution and then dried naturally.
b. Then, we prepared 200 mL of 2 mg/mL aqueous solution of dopamine hydrochloride. Tris-buffer was added into PDA solution to adjust pH to 8.5. After that, different amounts of AGQDs and a certain amount of PEI were added to the solution. The mixed solution was stirred at room temperature for 2 h to obtain the homogeneous aqueous solution in the co-deposition reaction.
c. We fixed the PES substrate on a stirred cell ultrafiltration device (Millipore^®^ Amicon, Billerica, MA, USA); then, 100 mL of PDA/PEI/AGQDs mixed solution was added to perform pressurized filtration under the pressure of 0.2 MPa at 25 °C.
d. Afterward, the unreacted PDA on the membrane surface was washed with deionized water. After being dried at 50 °C for 15 min, the fabricated membrane was used for characterization and performance testing.

The prepared membranes definitions and the concrete co-deposition conditions are shown in Table 1.

### 2.4. Characterization of AGQDs and NF Membranes

The comprehensive characterizations of AGQDs by various methods, including Fourier transform infrared spectroscopy (FT-IR), transmission electron microscopy (TEM), atomic force microscopy (AFM), X-ray photoelectron spectroscopy (XPS), zeta potential, and X-ray diffraction (XRD) were based on our previous work [21]. The UV-Vis spectrophotometer (PerkinElmer Lambda 750 S, Massachusetts, USA) was used to obtain the UV-Vis absorption spectra of GQDs and AGQDs.

The functional groups and chemical composition of the membrane surfaces were analyzed by attenuated total reflectance-Fourier transform infrared spectroscopy (ATR-FTIR, Bruker Vertex 70, Karlsruhe, Germany) and XPS (Physical Instruments Quantum 2000, Newark, DE, USA), respectively. The membrane surface hydrophilicity was measured using a contact angle goniometer (Beijing HARKE SPCAX3, China). The microscopic morphology of the prepared membranes was observed by SEM (LEO-1530, Oberkochen,, Germany) operated at 10 kV.

### 2.5. Photocatalytic Performance of GQDs and AGQDs

The photocatalytic performance of GQDs and AGQDs on methyl blue (MB) was evaluated using a 75 W white LED lamp. Briefly, 1 mg of GQDs or AGQDs powder was dissolved in a 20 ppm MB aqueous solution. The resulting solution was placed in the dark for 30 min to reach adsorption–desorption equilibrium. After that, the mixture was irradiated under the LED lamp for 30 min, and the color change of the solution before and after irradiation was recorded. The absorption of MB solution before and after irradiation was measured by a UV-Vis spectrometer (SHIMADZU, UV-1780, Shimadzu, Japan), and the concentration was calculated by Lambert–Beer law. The photocatalytic abilities of GQDs or AGQDs were assessed in terms of the degradation degree of MB. The degradation degree (*D*) of MB is calculated by Equation (1):

(1) $$D\left(\%\right)\;=\;\left(1-\frac{C_{a}}{C_{b}}\right)\times \;100\%$$

where *C_b_* and *C_a_* refer to the MB concentration before and after irradiation, respectively.

### 2.6. Separation Performances of NF Membranes

Water flux and rejection are critical parameters of NF membrane performance. Four different dyes (MB, CR, MO, and RhB) were used to evaluate the overall dye rejection of fabricated NF membranes. A dead-end permeation cell ultrafiltration device (Millipore^®^ Amicon, Billerica, MA, USA) was used to measure flux and rejection under the pressure of 2 bar at 25 °C.

The water flux is calculated by the following Equation (2):

(2) $$J=\frac{V}{A\Delta t}$$

where *J* is the water flux (L·m^−2^·h^−1^, LMH), *V* is the permeate volume (L), *A* represents the effective membrane area (m^2^), and ∆*t* is the operation time (h).

The dye rejection (R) is calculated by the following Equation (3):

(3) $$R\left(\%\right)\;=\;\left(1-\frac{C_{P}}{C_{c}}\right)\times \;100\%$$

where *C_p_* and *C_c_* represent the dye concentration of the permeate and the concentrate, respectively. The UV spectrophotometer was used to determine the dye concentration based on the standard curve between the absorbance and the dye concentration.

### 2.7. Photocatalytic Self-Cleaning Performance of NF Membranes

**Figure 2:**
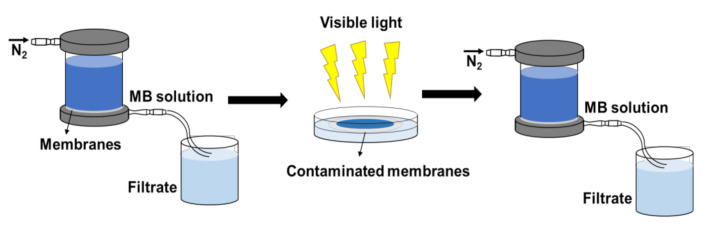
Self-cleaning test process of NF membrane.

The photocatalytic self-cleaning performance of the tested membranes was evaluated in terms of membrane flux and dye rejection before and after light irradiation. The self-cleaning test process is illustrated in Figure 2. First, the tested membrane was installed and continuously filtered by MB solution for 8 h, recording flux and MB rejection each 20 min. Subsequently, the fouled membrane was washed with deionized water. After that, the washed membrane was irradiated under LED white light for 3 h. Finally, the flux and rejection of the membrane was re-measured after light irradiation.

## 3. Results and Discussion

### 3.1. UV-Vis Diffuse Reflectance Characterization of GQDs and AGQDs

**Figure 3:**
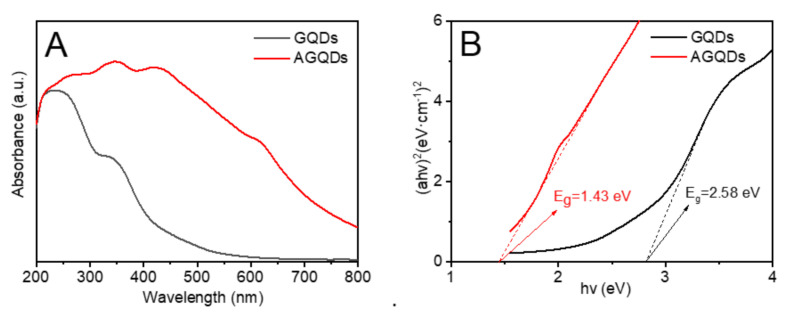
(**A**) UV—Vis spectra, (**B**) corresponding (*ahv*)^2^ versus h*v* plots of GQDs and AGQDs.

The AGQDs synthesized by the bottom–up method have a dot-like structure with 4–9 nm (Figure 1). The comprehensive characterizations of AGQDs have been reported in our previous study [21]. The optical response of GQDs and AGQDs can be evaluated by UV-Vis diffuse reflectance spectroscopy. Compared with GQDs, the absorption peak of AGQDs shows a significant redshift, and the tail extends to the visible region. It indicates that AGQDs can absorb a part of visible light [15]. The bandgap energy can be obtained from the (αhν)^2^ versus hν plots according to the Kubelka–Monk rule [15]. As shown in Figure 3B, the bandgap energy of AGQDs (1.43 eV) is significantly lower than that of GQDs (2.58 eV). The lower bandgap energy of AGQDs reflects the stronger visible light response of AGQDs, which enhances the photocatalytic performance of AGQDs [11]. The edge-connected aspartic acid of AGQDs may introduce n-orbitals between π and π* orbitals, which leads to the lower bandgap energy of AGQDs [18,22].

### 3.2. Characterization of the Prepared NF Membranes

**Figure 4:**
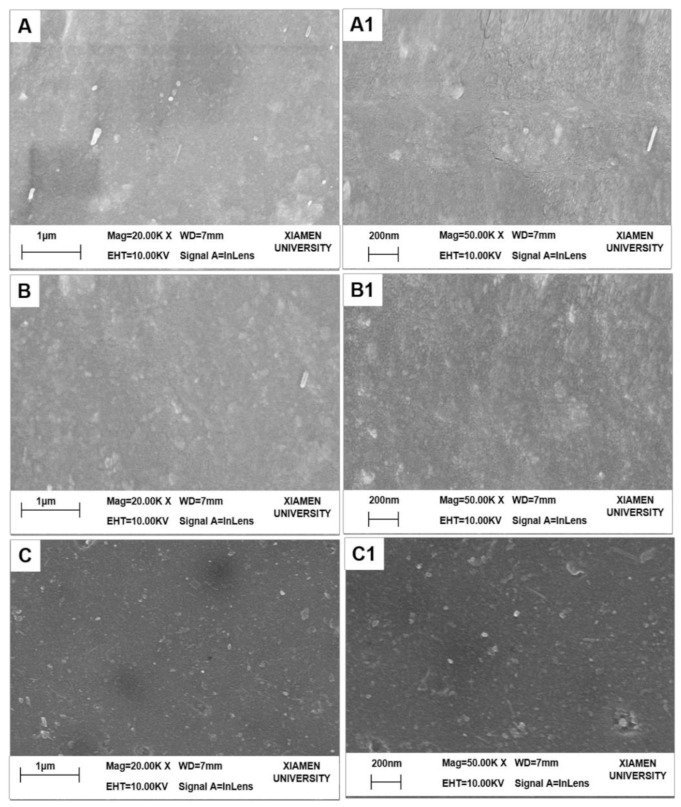
SEM images of UF and NF membranes (**A**–**C**: 20.00 K×, **A1**–**C1**: 50.00 K×): (**A**) PES substrate, (**B**) M3 membrane, (**C**) M3/AGQDs-0.2 NF membrane.

The surface morphologies of the fabricated NF membranes are shown in Figure 4. The PES substrate, M3 (PDA/PEI-10000), and M3/AGQDs-0.2 membranes show different morphological structures. Compared to the smooth surface of the PES substrate, the M3 membrane exhibits a rougher surface due to the disordered stacking of PDA and PEI during the co-deposition process [23,24]. The non-covalent interaction of PDA and PEI via co-deposition would produce an unstable PDA/PEI top layer [25]. It is reported that introducing reactive oxygen species (ROS) contributes to generating a more stable cross-linked structure of PDA, forming a tight selective layer [20,26]. As shown in Figure 4C, the surface structure of the M3/AGQDs-0.2 membrane became smooth after the introduction of AGQDs, which may be due to the fact that AGQDs also produce ROS (O_2_^−^ and·OH^−^) after irradiation [15]. It suggests that AGQDs contributed to the chemical cross-linking of PDA and PEI to form a more compact, smooth, and stable membrane structure.

**Figure 5:**
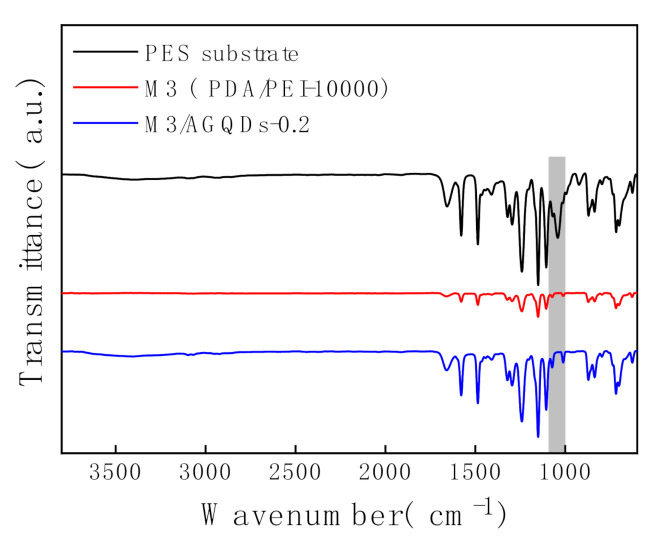
ATR—FTIR spectra of NF membranes.

The chemical compositions of the membrane surfaces are analyzed by ATR-FTIR, as shown in Figure 5. The PES substrate exhibits a more pronounced characteristic peak around 1000–1100 cm^−1^, which is attributed to the stretching vibration of the -SO_2_- group. In contrast, the intensities of the absorption peaks at 1000–1100 cm^−1^ for the M3 membrane and the M3/AGQDs-0.2 membrane are significantly weakened, which indicates that the M3/AGQDs-0.2 layer has been successfully deposited on the top of the PES substrate.

**Figure 6:**
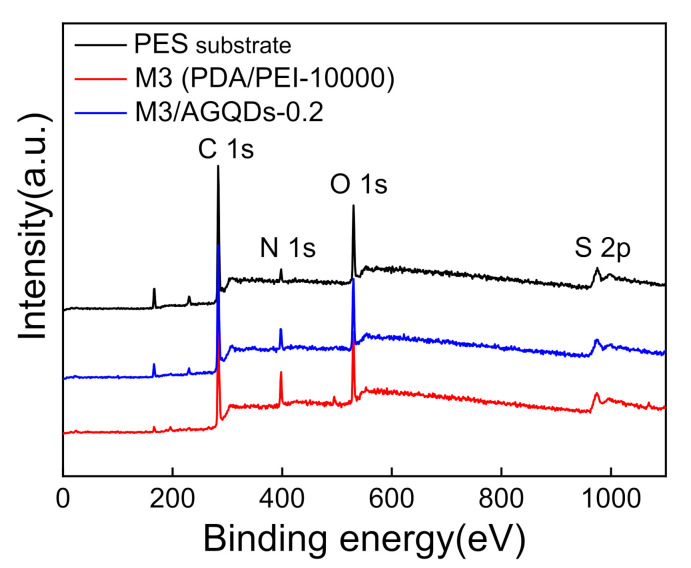
XPS full spectra of PES substrate and NF membranes.

XPS was used to further analyze the chemical compositions and elemental contents of the membrane surfaces. According to Figure 6 and Table 2, the PES substrate presents the highest S content owing to the existence of the S element in the PES structure. Meanwhile, the M3 and the M3/AGQDs-0.2 membranes have the lower S element because of the formation of a thin selective layer on the PES substrate. This is consistent with the ATR-FTIR results, demonstrating the deposition of selective layers on the PES substrate. It is found that the PES substrate contains a N element from its XPS spectra, which probably derives from the residual pore former such as polyvinylpyrrolidone containing a N element. In addition, according to Table 2, the M3/AGQDs-0.2 membrane has the highest N content, which is attributed to the abundant N element from PEI and AGQDs [21]. The introduction of AGQDs might improve the degree of cross-linking between PDA and PEI during the co-deposition process, as deduced by the following mechanism analysis between PEI and PDA.

**Figure 7:**
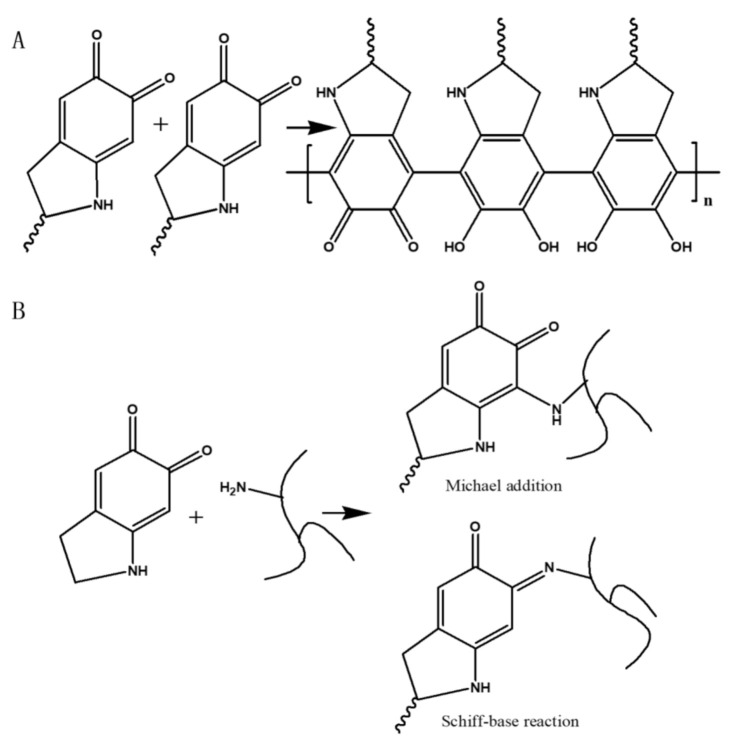
Reaction mechanisms between PDA and PEI: (**A**) self-polymerization of PDA, (**B**) Michael addition and Schiff base reaction of PDA and PEI.

The reaction mechanism of PDA and PEI is shown in Figure 7. It has been reported that the pH has a significant effect on the reaction between PDA and amines [27]. When the pH is less than 8.5, it is difficult for the amino group to react with the catechol group inside PDA [28]. However, when pH is high above 8.5, the catechol group of PDA can be oxidized to form quinone groups, which can more easily react with the amino group via Michael addition or Schiff base reaction [29]. Moreover, the self-polymerization of PDA in alkaline solutions contributes to the adhesion of PDA to the PES substrate. In this study, the pH of the PDA solution was adjusted to 8.5 by tris-buffer solution. As a result, PEI was more readily chemically cross-linked with PDA, which improved the structural stability of the PDA/PEI composite.

**Figure 8:**
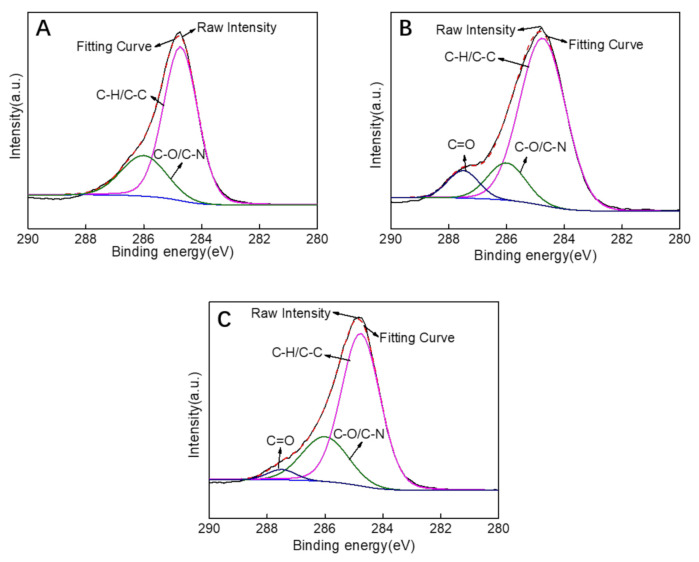
XPS C1s spectrum of PES substrate (**A**), M3 (PDA/PEI-10000) membrane (**B**), M3/AGQDs-0.2 membrane (**C**).

The fitting curves were obtained by deconvolution of the raw intensity of XPS C1s for the PES substrate, M3 (PDA/PEI-10000), and M3/AGQDs-0.2 membranes (Figure 8A–C). The characteristic peak at 287.5 eV for the M3 and M3/AGQDs-0.2 membranes is attributed to C=O in PDA due to the quinoid group within PDA [30]. Compared to the M3/AGQDs-0.2 membrane, the M3 membrane shows a strong C=O characteristic peak. It is attributed to the introduction of AGQDs generating ROS [31], which promotes the Schiff base reaction of PDA and reduces C=O groups content. It is worth noting that AGQDs can be involved in cross-linking reactions being covalently attached to the PDA/PEI/AGQDs selective layer. The characteristic peak at 286 eV is attributed to the C–O/C–N group. The C–N groups in the prepared membranes are mainly derived from the self-polymerization of PDA, chemical cross-linking of PDA/PEI, and additional introduction of AGQDs. The intensity of the C–O/C–N characteristic peak of the M3/AGQDs-0.2 membrane is stronger than that of the M3 membrane. On the one hand, the AGQDs in the PDA/PEI/AGQDs selective layer introduce an extra N element. On the other hand, the –NH_2_ from AGQDs can participate in the cross-linking reaction, increasing the C–N content. The results further demonstrate that AGQDs have a significant effect on the chemical cross-linking of PDA and PEI.

**Figure 9:**
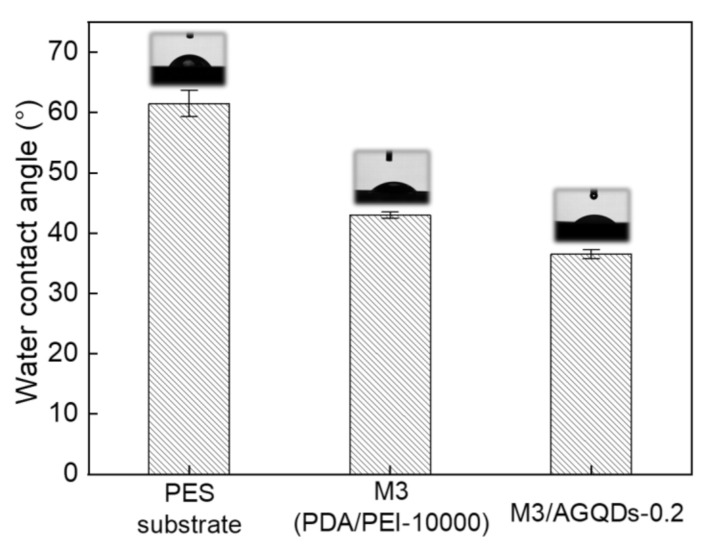
The water contact angle of the prepared membranes.

The water contact angle (WCA) of the NF membrane is closely related to the roughness and hydrophilicity of the membrane surface, as shown in Figure 9. It was found that the introduction of the PDA/PEI selective layer significantly reduced the hydrophilicity of the membrane surface, with a decrease in WCA from 61.5° to 43.0°. The AFM results show that the PES substrate has a similar surface roughness to the M3 membrane. Therefore, the remarkably decreasing WCA of the M3 membrane benefits from the excellent hydrophilicity of PDA [32]. The M3/AGQDs-0.2 membrane demonstrates the lowest WCA (36.5°), which is caused by the synergistic effects of the smooth membrane surface and the inherent hydrophilic properties of AGQDs and PDA.

### 3.3. Separation Performance of the Fabricated Membranes

**Figure 10:**
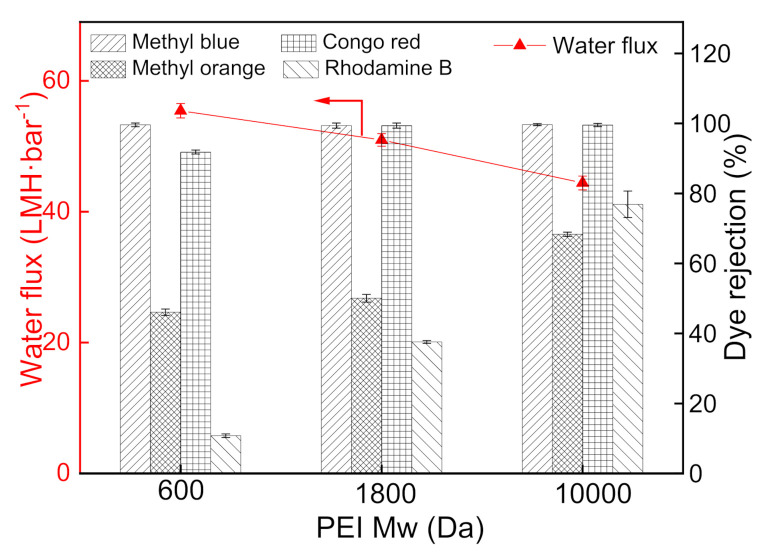
Influence of PEI molecular weight on the separation performance of PDA/PEI membranes (testing conditions: 2 bar, 25 °C).

The PEI molecular weight for constructing the PDA/PEI membranes performance was optimized from three kinds of PEI molecular weights (600, 1800, and 10,000 Da) [33,34]. According to Figure 10, the water flux of the prepared membrane is inversely proportional to the PEI molecular weight, owing to the higher molecular weight PEI cross-linking with the PDA to form a denser selective layer. Correspondingly, the increased molecular weight of PEI contributes to the improved dye rejection of the PDA/PEI membranes. It is found that PEI with a molecular weight of 10,000 Da exhibits a high rejection with the reasonable flux, which is regarded as the optimal molecular weight of PEI cross-linking reagent in this study.

**Figure 11:**
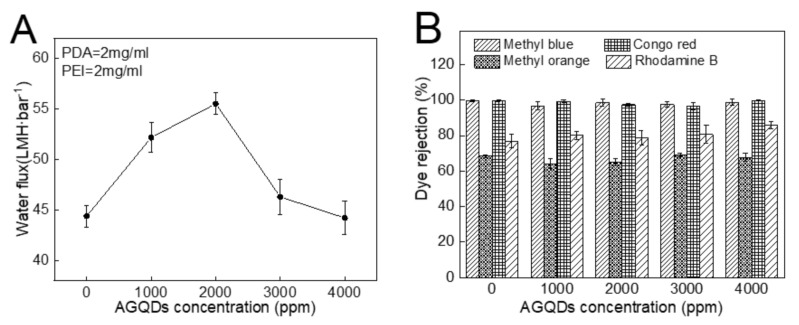
Water flux (**A**) and dye rejections (**B**) of PDA/PEI/AGQDs membranes as a function of AGQDs concentration.

The influence of the AGQDs concentrations on the separation performance of the PDA/PEI/AGQDs membranes is shown in Figure 11. With the increase in AGQDs concentration, the water flux shows a trend of first increasing and then decreasing. Compared to the M3 (PDA/PEI-10000) membrane, introducing a suitable amount of AGQDs contributes to optimizing the membrane microstructures and properties, which improves the water flux without reducing dye rejection. On the one hand, AGQDs can facilitate the cross-linking reaction between PDA and PEI. On the other hand, the presence of AGQDs may reduce the membrane pore blockings caused by PDA self-polymerization [35]. However, while AGQDs content increases from 2000 to 4000 ppm, the water flux of the PDA/PEI/AGQDs membranes decreases from 55.5 to 44.2 LMH·bar^−1^. The water flux reduction is excessive, since AGQDs may block the pore channels and increase the filtration resistance. Therefore, considering the permeability and rejection of NF membranes, the optimum concentration of AGQDs is 2000 ppm to construct the PDA/PEI/AGQDs membrane.

Four kinds of dyes (MB, CR, MO, and RhB) with different molecular weights and charges are used to systematically investigate the separation performance of the PDA/PEI/AGQDs membranes [13]. The molecular weights and charge properties of different dyes are listed in Table 3, and the dye rejections of the M3/AGQDs membranes are shown in Figure 11B. The M3/AGQDs membranes present almost 100% rejection toward MB and CR, primarily resulting from the size exclusion effect between the PDA/PEI/AGQDs membranes and dyes with large molecular weight (MB and CR). The rejection of four dyes follows the order of MB ≈CR > RhB > MO, which is similar to the molecular weight order of four dyes. It indicates that the size exclusion effect plays a critical role in dye rejection.

### 3.4. Self-Cleaning Performance of the Fabricated Membranes

The anti-fouling performance is crucial for the successful application of NF membranes [36]. Severe membrane fouling will significantly increase the cost of water treatment [37]. The engineered NF membrane endowed with photocatalytic self-cleaning property is expected to improve the anti-fouling performance [38]. In this study, the photocatalytic self-cleaning performance of the M3/AGQDs-0.2 membrane was evaluated by 8 h continuous filtration.

**Figure 12:**
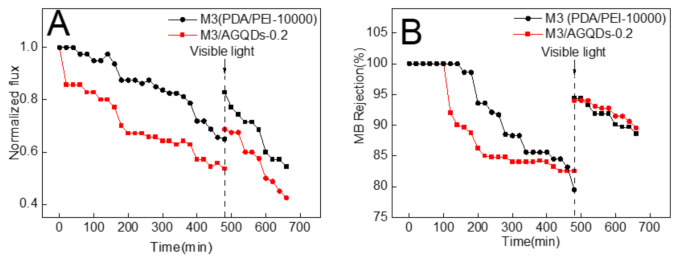
Photocatalytic self-cleaning properties of the prepared membranes: (**A**) normalized water fluxes and (**B**) MB rejection before and after irradiation.

MB has a large molecular weight and a high rejection rate, which results in its easy enrichment on the membrane surface. Thus, MB was selected as a mode foulant to evaluate the self-cleaning performance of the selected membranes (the M3 and M3/AGQDs-0.2 membranes). Figure 12 shows the normalized flux and MB rejection before and after light irradiation. According to Figure 12A, the normalized fluxes of the M3 and M3/AGQDs-0.2 membranes gradually decreased with the operation time. On the one hand, the enrichment of retained MB molecules on the membrane surface may cause concentration polarization [39]. On the other hand, MB could block the membrane pores, which increased the mass transfer resistance of the membrane filtration process [40]. As a result, the synergistic effect leads to a significant flux reduction. The water flux could be partially restored after water washing and light irradiation. Most importantly, the introduction of AGQDs imparts photocatalytic degradation ability to the M3/AGQDs-0.2 membrane. The M3/AGQDs-0.2 membrane shows a significantly higher flux recovery rate than the M3 membrane (83% vs. 69%) owing to the photocatalytic degradation of MB by the M3/AGQDs-0.2 membrane. In contrast, the M3 membrane only removed a limited number of foulants from the membrane surface by water washing, resulting in a lower flux recovery rate.

The MB rejection of the NF membranes mainly depends on by the size sieving effect and the Donnan effect. As shown in Figure 12B, in the early filtration stages, the tested NF membranes have a high MB rejection under the combined influences. With more and more MB enrichment on the membrane surface, the Donnan effect gradually weakens, which results in a lower rejection. After water washing and light irradiation, the MB rejection of the M3/AGQDs-0.2 membrane recovers to the initial level.

**Figure 13:**
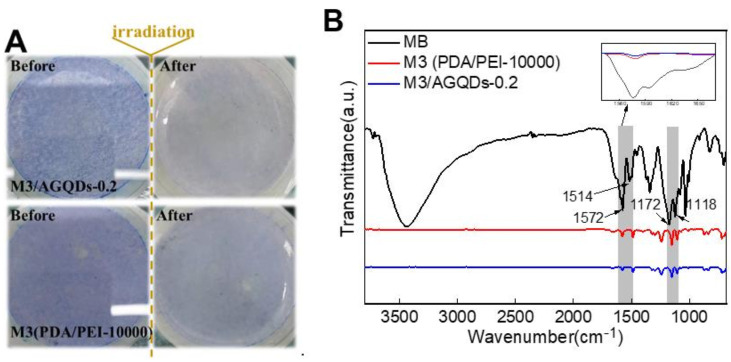
The surface of NF membranes before and after visible light irradiation (**A**) and FT—IR spectrum of methyl blue and NF membranes (**B**).

Figure 13A shows the color variation of the membrane surface before and after light irradiation. It is obvious that the M3/AGQDs-0.2 membrane exhibits better photocatalytic self-cleaning performance than the M3 (PDA/PEI-10000) membrane. The blue color of MB almost disappears from the M3/AGQDs-0.2 membrane surface after light irradiation, while the blue color remains on the surface of the PDA/PEI membrane. After light irradiation, ATR-FTIR was used to further measure the residual MB on the membrane surface. Figure 13 demonstrates the FTIR spectra of the MB powder, M3, and M3/AGQDs-0.2 membranes surface after irradiation, respectively. The aromatic skeleton absorption peak of MB is found at 1514–1572 cm^−1^. The absorption peaks at 1172–1118 cm^−1^ are attributed to the symmetric and asymmetric stretching vibrations of the sulfonic acid group S=O in MB [41]. According to Figure 13B, the M3/AGQDs-0.2 membrane exhibits a remarkably weaker characteristic peak of MB than the M3 membrane. This result further indicates that the M3/AGQDs-0.2 membrane demonstrates excellent photocatalytic degradation toward MB with the help of AGQDs.

**Figure 14:**
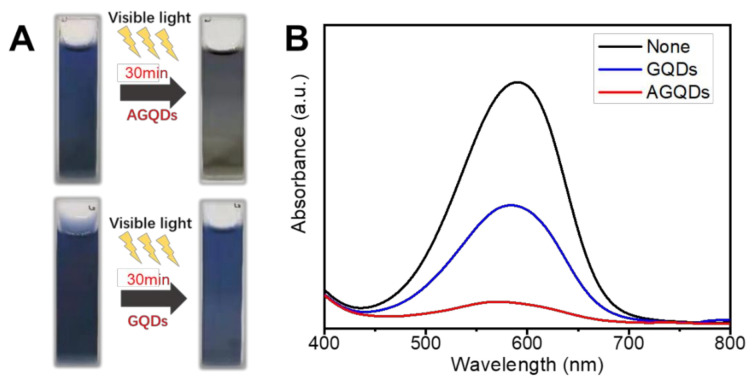
Photocatalytic degradation of MB by AGQDs and GQDs (**A**), UV-Visible spectrum of MB after irradiation (**B**).

The photocatalytic degradation mechanism of MB by AGQDs is explored by comparison with GQDs. According to Figure 14A, both AGQDs and GQDs exhibit photocatalytic abilities for MB. Under visible light conditions, the valence electrons (e^−^) in the photocatalyst can be excited to the conduction band (CB) and generate ROS (such as O_2_^−^ and ·OH^−^) clusters in the aqueous environment. ROS can combine with MB and carry out redox reactions to produce CO_2_, H_2_O, and degradation products [15,42,43].

According to Figure 14B, the photocatalytic degradation of MB by AGQDs was significantly better than that of GQDs. Compared with GQDs, the concentration of MB solution with AGQDs is significantly decreased after light irradiation (MB degradation rate 88% vs. 51%), which is consistent with the color change results in Figure 14A. According to the results in Figure 3B, AGQDs exhibit a lower bandgap energy than GQDs, indicating that AGQDs have a better visible light absorption capacity [44]. The low bandgap energy allows AGQDs to produce more photogenerated charge carriers (e^−^ and h^+^) in visible light, thereby facilitating MB degradation. In addition, as a photosensitizer, MB turns into an unstable excited state under visible light irradiation. The oxygen atoms with strong oxidation properties are generated in the excited state, which leads to the spontaneous degradation process. In the presence of AGQDs, the e^−^ generated by MB excitation can be stored in AGQDs, which may hinder the complexation between e^−^ and h^+^ and hence be more favorable for MB degradation. Additionally, Table 4 indicates that the optimal PDA/PEI/AGQDs membrane in this study achieves higher water flux and dye rejection than other NF membranes in the literature. Therefore, the co-deposition method can be an effective strategy for constructing high-performance dye separation membranes.

## 4. Conclusions

In this study, the PDA/PEI/AGQDs NF membrane with self-cleaning performance was constructed by the co-deposition method. The results indicate that the introduction of photocatalytic AGQDs into the selective layer contributes to improving the separation performance and imparts photocatalytic self-cleaning performance to NF membranes. The PDA/PEI/AGQDs membrane fabricated by suitable PEI molecular weight (10,000 Da) and AGQDs concentration (2000 ppm) demonstrated the highest water permeability of 55.5 LMH·bar^−1^ while maintaining high dye rejection (99.7 ± 0.3% for MB). This is achieved by the formation of the dense and hydrophilic PDA/PEI/AGQDs selective layer. Moreover, the M3/AGQDs-0.2 membrane demonstrates excellent self-cleaning performance due to the low bandgap energy of AGQDs. The water flux recovery of the M3/AGQDs-0.2 membrane is significantly higher than that of the M3 (PDA/PEI-10000) membrane after light irradiation. Generally, through in-depth analysis of the degradation MB mechanism of AGQDs, the excellent photocatalytic degradation performance of AGQDs is well confirmed, which provides a new approach to construct self-cleaning NF membranes.

## Figures and Tables

**Table 1:** The co-deposition reaction conditions of TFC and TFN NF membranes.

Membranes	PDA Concentration (% *w/v*)	PEI Mw (Da)	PEI Concentration (% *w/v*)	AGQDs (ppm)	Temperature of Heat Treatment (°C)
M1	0.2	600	0.2	0	50
M2	0.2	1800	0.2	0	50
M3	0.2	10,000	0.2	0	50
M3/AGQDs-0.1	0.2	10,000	0.2	1000	50
M3/AGQDs-0.2	0.2	10,000	0.2	2000	50
M3/AGQDs-0.3	0.2	10,000	0.2	3000	50
M3/AGQDs-0.4	0.2	10,000	0.2	4000	50

**Table 2:** The chemical compositions of PES substrate and NF membranes by XPS.

Membranes	Atomic Concentration (%)				^a^ O/N	^b^ O/C
	C	N	O	S		
PES substrate	74.72	3.53	17.68	4.07	1.29	0.23
M3 (PDA/PEI-10000)	71.85	7.92	17.66	2.57	1.30	0.24
M3/AGQDs-0.2	68.85	12.42	17.53	1.20	1.41	0.25

^a^ O/N: molar ratio of nitrogen to carbon in the selected layers.; ^b^ O/C: molar ratio of oxygen to carbon in the selected layers.

**Table 3:** Molecular weight and charged property of different dyes.

Dyes	Methyl Blue	Methyl Orange	Congo Red	Rhodamine B
Molecular weight [g·mol^−1^]	799.80	327.33	696.68	479.01
Charge under pH = 7.0	positive	negative	negative	positive

**Table 4:** Comparisons of water permeability and MB rejection between this study and literatures.

Membrane Type	Permeability (LMH/bar)	Testing Conditions	MB Rejection (%)	Reference
CuTz-1-GO/PAN	40.2	500 ppm, 0.4 Mpa, 25 °C	94.9	[45]
TA-Fe^3+^/PAN	40.9	35 µmol/L, 0.2 Mpa, 25 °C	93.9	[46]
GO-Ca-SA/PVDF	38.9	20 ppm, 0.12 Mpa, 25 °C	99+	[47]
MXene-PEI-TMC/PAN	20.9	200 ppm, 0.4 Mpa, 25 °C	98.84	[48]
TiO2-HMDI/PES	26	35 µmol/L, 0.2 Mpa, 25 °C	99.1	[49]
PIP-CS-TMC/PES	49.6–128.8	100 ppm, 0.5 Mpa, 25 °C	99+	[50]
ZIF-8&PEI/PAN	33.0	100 ppm, 0.4 Mpa, 20 °C	99.6	[51]
AGQDs-PDA-PEI/PES	55.5	20 ppm, 0.2 Mpa, 25 °C	99.7	This work

## Data Availability

The data presented in this study are available on request from the corresponding author.
